# A new family of nitrate/nitrite transporters involved in denitrification

**DOI:** 10.1007/s10123-018-0023-0

**Published:** 2018-07-20

**Authors:** Laura Alvarez, Dione Sanchez-Hevia, Mercedes Sánchez, José Berenguer

**Affiliations:** 1 Centro de Biología Molecular Severo Ochoa, Universidad Autónoma de Madrid- Consejo Superior de Investigaciones Científicas, 28049 Madrid, Spain; 2 Present Address: Laboratory for Molecular Infection Medicine Sweden, Department of Molecular Biology, Umeå University, 90187 Umeå, Sweden

**Keywords:** *Thermus*, Denitrification, Nitrate, Nitrite, Transport, MFS

## Abstract

Denitrifying bacteria carry out nitrate and nitrite respiration inside and outside the cell, respectively. In *Thermus thermophilus*, nitrate and nitrite transport processes are carried out by major facilitator superfamily (MFS) transporters. The sequence of the *nar* operon of nitrate-only respiring strains of *T. thermophilus* includes two tandemly organized MFS transporter genes (*narK* and *narT*) of the NarK1 and NarK2 families. Both can function as nitrate/nitrite antiporters, but NarK has been proposed as more specific for nitrate whereas NarT more specific for nitrite. In some nitrate- and nitrite-respiring strains of the same species, a single MFS transporter (NarO) belonging to a different MFS subfamily appears. To analyze the role of this single MFS in the same genetic context, we transferred the two types of *nar* operon to the aerobic strain HB27, and further included in both of them the ability to respire nitrite. The new denitrifying strains HB27dn, with two MFS, and HB27dp, with a single one, were used to isolate mutants devoid of transporters. Through *in trans* complementation experiments, we demonstrate that the NarO single MFS works efficiently in the transport of both nitrate and nitrite.

**Electronic supplementary material:**

The online version of this article (10.1007/s10123-018-0023-0) contains supplementary material, which is available to authorized users.

## Introduction

The use of nitrate either as substrate for nitrogen assimilation or as electron acceptor under anaerobic conditions requires its active transport through the cytoplasmic membrane to reach the concentrations required for the assimilative or respiratory nitrate reductases to function. These reductases produce nitrite which cannot accumulate due to its toxicity for the cells. Nitrite elimination can be achieved through the activity of the assimilatory nitrite reductase, which produces ammonium, or through its extrusion by specific transporters in the case of nitrate respiration, as further steps in the denitrification pathway are catalyzed in the periplasm.

In most denitrifying bacteria, nitrate and nitrite transport processes are carried out by transmembrane transporters belonging to the major facilitator superfamily (MFS) of proteins, forming a distinct subfamily known as NarK (Clegg et al., 2002; Moir & Wood, 2001; Pao et al., 1998). Like all MSF members, NarK transporters generally include 12 transmembrane-spanning helices, where helices 1, 2, 4, 5, 7, 8, 10, and 11 form the central transport pore. Sequence comparisons have revealed the existence of two major NarK types of proteins, where NarK1 subtype clusters with proteins linked to nitrate assimilation operons, whereas the NarK2 subtype includes putative nitrate/nitrite antiporters associated to nitrate reductase operons (Goddard et al., 2017; Moir & Wood, 2001; Wood et al., 2002). To this latter type belong the NarU and NarK proteins of *E. coli* for which structural X-ray models are available (Yan et al., 2013; Zheng et al., 2013). However, whereas NarK has been suggested to function as a nitrate/nitrite antiporter, the NarU homolog is proposed to function as a nitrate/cation symporter, showing that there is not a clear-cut way to define the actual role of these transporters even knowing their 3D structure.

In some denitrifying bacteria, like *Pseudomonas aeruginosa* PA01 (Sharma et al., 2006) or in nitrate respiring strains of *Thermus thermophilus* (Ramirez et al., 2000), two NarK proteins, one of the NarK1 and the other of the NarK2 subtypes, appear encoded in tandem near to or as part of the operon for the respiratory nitrate reductase. In other bacteria like *Pseudomonas denitrificans* or *Paracoccus denitrificans*, these two proteins are fused in a single polypeptide that contains 24 alpha-helices divided in two domains that have been shown to be functional when expressed separately, despite some apparent interdependence has been detected (Goddard et al., 2008). Either as separate proteins or as a single fusion, the first protein or protein domain belongs to the NarK1 subtype of MFS transporters, whereas the second belongs to the NarK2 subtype. Recently, it has been proven by complementation assays in *Paracoccus denitrificans* that the NarK1-like domain of a fusion NarK1-NarK2 protein functions basically as nitrate transporter but that it is still able to function as nitrate/nitrite symporter, whereas the NarK2-like domain is more specialized in nitrate/nitrite antiport (Goddard et al., 2017).

Thermophilic organisms have to deal with low oxygen availability due to its low solubility at the high temperatures of their natural habitats. Actually, even in thermophilic genera described as strictly aerobes like *Thermus thermophilus*, horizontally transferable genetic islands exist that encode for the ability to denitrify (Alvarez et al., 2014). In fact, many natural isolates of this species are able to grow anaerobically by nitrate respiration with the accumulation of nitrite as the final respiration product, whereas other strains of the same species can further reduce nitrite to produce water insoluble N_2_O (Alvarez et al., 2014; Cava et al., 2008).

The last two genes of the nitrate respiration operon (*narCGHJIKT*) of the *T. thermophilus* NAR1 strain encode NarK1- and NarK2-like transporters, re-named as NarK and NarT, respectively. Mutants of this strain lacking both proteins cannot grow anaerobically with nitrate (Ramirez et al., 2000), thus supporting the absence of alternative transporters for nitrate under these conditions. Although with different efficiencies, single *narT* or *narK* deletion mutants can grow anaerobically with nitrate and also accumulate nitrite upon addition of nitrate to the growth medium, supporting that both of them can function as nitrate/nitrite antiporters, despite a major role for NarT (NarK2) in this antiporter activity was deduced from its higher phenotypic affection (Ramirez et al., 2000).

Unexpectedly, the genomes of some recently sequenced denitrifying strains of *Thermus* spp. encode a single member of the NarK family, named NarO, which shows a low identity towards NarK1 or NarK2 subfamilies and actually clusters in a separate group of MSF transporters. In this work, we analyze the role of the NarO protein in comparison to the in tandem NarK1–2 genes in the same genetic context. Our data show that the NarO protein can replace the NarK1 and NarK2 types of transporters, resulting in restoration of the wild-type phenotype in a nitrate/nitrite transporter defective mutant. These data support that the NarO type of nitrate transporters are actually flexible bifunctional nitrate/proton symporters and nitrate/nitrite antiporters, and suggest that the presence of two transporters may constitute a selective trait to avoid nitrite toxicity in those strains in which this nitrogen oxide constitutes the final product of anaerobic respiration.

## Materials and methods

### Bacterial strains and growth conditions

The strains of *Thermus thermophilus* used in this work are indicated in Table 1. *T. thermophilus* HB27 grows only under areobic conditions, whereas strains *T. thermophilus* NAR1 and PRQ25 can grow anaerobically by nitrate respiration and denitrification respectively (Cava et al., 2008). The strains *T. thermophilus* HB27dn and HB27dp are denitrifying derivatives of HB27 that encode the nitrate respiration cluster from the NAR1 strain (HB27dn) or from the PRQ25 strain (HB27dp) and the nitrite respiration cluster (*nic*) from the PRQ25 strain (Alvarez et al., 2011). For aerobic growth, the strains were incubated on TB medium (Ramírez-Arcos et al., 1998) at 60 °C with mild shaking (150 r.p.m.). Anaerobic growth was achieved in Eppendorf tubes containing 2 ml of TB medium supplemented with potassium nitrate (20 mM) overlaid by mineral oil and incubated statically at 60 °C. Agar (1.5% *w*/*v*) was added to the TB medium for growth on plates, which were incubated at 60 °C in a water-saturated atmosphere. *Escherichia coli* DH5α [*supE44 Δ(lacZYA-argF)U169* Φ80*lacZΔM15 hsdR17 recA1 endA1 gyrA96 thi-1 relA1*] was used for plasmid construction. *E. coli* was routinely grown at 37 °C in liquid or solid LB medium. Ampicillin (100 μg/ml), kanamycin (30 μg/ml), or hygromycin B (100 μg/ml) were added when needed.

**Table 1:** *Thermus thermophilus* strains used in this work

Strain name	Phenotype and genotype	Reference or source
HB27	Wild type aerobic	Dr. Koyama
NAR1	Nitrate respiring	(Cava et al., 2008; Vieira & Messing, 1982)
PRQ25	Denitrifying	(Manaia et al., 1994)
HB27dn	Denitrifying with NCE from NAR1, NarK, and NarT transporters	(Alvarez et al., 2011)
HB27dp	Denitrifying with NCE from PRQ25, single NarO transporter	(Alvarez et al., 2011)
HB27dn ∆*narKT::kat*	HB27dn devoid of transporters, Kan^R^	This work
HB27dn ∆*narKT*	HB27dn devoid of transporters	This work
HB27dp ∆*narO::kat*	HB27dp devoid of transporters, Kan^R^	This work
HB27dp ∆*narO*	HB27dp devoid of transporters	This work

### Sequence analysis and DNA methods

A draft sequence from *T. thermophilus* PRQ25 was obtained through pyrosequencing in a Roche-454 system (Lifesequencing, Valencia, Spain). The genes encoding enzymes implicated in the denitrification process were identified in the contigs by BLAST sequence comparisons (Alvarez et al., 2011). The sequence of the *nar* operon of this strain has been deposited in the GenBank database with the accession number MH158735.

### Phylogenetic comparisons

Homolog search was performed with *BLAST* (Altschul et al., 1990) against a non-redundant protein database. A protein data set of 34 sequences was aligned using CLUSTAL OMEGA version 1.2.4 (Sievers et al., 2011). The data set includes reference sequences from *E. coli*, *P. aeruginosa*, or *P. denitrificans.* For visualization of the generated tree, the Interactive Tree of Life (iTOL) v3 tool was used (Letunic & Bork, 2016). Protein sequences used in this analysis are listed in supplemental Table S1.

### DNA techniques

Plasmid purification, restriction analysis, plasmid construction, polymerase chain reaction (PCR), and routine DNA sequencing were carried out by standard methods (Sambrook et al., 1989). Plasmids used are listed in Table 2.

**Table 2:** Plasmids used in this work

Plasmid	Description or use	Reference or source
pKT1	Source of *kat* cassette	(Lasa et al., 1992)
pUC119	Suicide plasmid in *Thermus*	(Vieira & Messing, 1982)
pUCΔnarKT	Mutation of *narKT*	This work
pUCΔnarKT::kat	Mutation of *narKT*	This work
pUCΔnarO	Mutation of *narO*	This work
pUCΔnarO::kat	Mutation of *narO*	This work
pMK184	Expression plasmid in *Thermus*	(Cava et al., 2007)
pMK184narK	Expression of NarK	This work
pMK184narT	Expression of NarT	This work
pMK184narO	Expression of NarO	This work
pMH184	Expression plasmid in *Thermus*	(Cava et al., 2007)
pMH184narK	Expression of NarK	This work
pMH184narT	Expression of NarT	This work
pMH184narO	Expression of NarO	This work

### Construction of HB27-denitrifying derivatives 27dn and 27dp

Genomic DNA from *T. thermophilus* PRQ25 strain was isolated by standard procedures. For transformation, genomic DNA (200 ng) from the PRQ25 strain was added to 0.5 ml of exponential cultures (optical density at 550 nm = 0.2), allowing incubation at 60 °C to continue for 4 h with standard shaking. For the selection of the HB27dn strain, a derivative of the nitrate-respiring HB27c strain was used as recipient strain and selection was carried out under anaerobic conditions at 60 °C for 48 h with sodium nitrite (5 mM). Individual colonies were isolated on TB plates and subjected to nitrate reduction/nitrite consumption assays (Alvarez et al., 2011). Nitrite-respiring transformants were selected. For the selection of the HB27dp strain, the HB27 strain was transformed as above, and anaerobic growth selection was carried out with potassium nitrate (20 mM) as above. Upon the isolation of nitrate-respiring colonies from plates, a further transformation step was carried out with selection by nitrite respiration as described above.

The presence of the *nar* cluster from NAR1 in 27dn or from PRQ25 in 27dp was confirmed by amplification of the *narO* and *narKT* genes respectively. A scheme of the denitrification clusters of each of the new strains is shown in supplemental Fig. S1.

### Isolation and complementation of mutants

Mutation of *narO* or *narKT* was performed by homologous recombination with DNA constructions containing approximately 1000-bp-long flanking regions of each targeted gene. The upstream regions were amplified by PCR using the primers mutnarOKTupdir, mutnarOuprev, and mutnarKTuprev (Table 3), while primers drpArbsXbaIdir and drpBstopEcoRIrev were used for the amplification of the downstream regions (Table 3). The PCR products were sequentially cloned between the SalI-XbaI-EcoRI restriction sites of plasmid pUC119 (Vieira & Messing, 1982), an *Escherichia coli* plasmid that does not replicate in *T. thermophilus.* The kanamycin resistance *kat* cassette (Lasa et al., 1992) was inserted into the XbaI site, oriented downstream to avoid polar effects on the expression of the nitrate reductase complex. This way, the HB27dn Δ*narKT::kat* and the HB27dp Δ*narO::kat* mutants were obtained. Further derivatives were obtained in which the *kat* cassette was deleted through additional recombination with linearized DNA encoding the recombination arms without the *kat* cassette. Kanamycin-sensitive mutant strains HB27dn Δ*narKT* and HB27dp Δ*narO* were obtained (Supplemental Fig. 1). Transformation and mutant selection were carried out as described (Alvarez et al., 2011). All the mutants isolated were checked by PCR.

**Table 3:** Oligonucleotides used in this work

Oligonucleotide	Sequence (5′-3′)	Purpose
katXbaIdir	AAAATCTAGACCCGGGAGTATAACAGA	Kat cassette
katXbaIrev	AAAATCTAGACGTTCAAAATGGTATGCGTTTTGA	Kat cassette
mutnarOKT up dir	AAAAGTCGACAGCTCTACACTCGCACCCT	pUCΔnarO::kat, pUCΔnarKT::kat
mutnarO up rev	AAATTCTAGATCAAAGGGTCTCCCGCC	pUCΔnarO::kat
mutnarKT up rev	AAATTCTAGATCACCTCGCCAGCTTGC	pUCΔnarKT::kat
drpArbsXbaIdir	AAAATCTAGAGGGCCTAGGGA	pUCΔnarO::kat, pUCΔnarKT::kat
drpBstopEcoRIrev	AAAAGAATTCCTAGCCCCCCT	pUCΔnarO::kat, pUCΔnarKT::kat
narOdir	CCAACCTTTCGGGCTCCCTC	Check narO
narOrev	CCGAGGGCGTAGAAGACGAG	Check narO
narTdir	CTTGGCGCACCCTCTGGATC	Check narKT
narTrev	GTACCCCGAGAAGTGCCCTC	Check narKT
narKrbsXbaIdir	AAAATCTAGACGAGGTGAGCTATGATCCAC	Overexpression NarK, pMK, and pMHnarK
narKstopEcoRIrev	AAATGAATTCTCAGCATGGACGGTCTCCTT	Overexpression NarK, pMK, and pMHnarK
narTrbsXbaIdir	AAAATCTAGAGGAGACCGTCCATGCTGAAG	Overexpression NarT, pMK, and pMHnarT
narTstopEcoRIrev	AATTGAATTCTTAGCAGGGCTTCTCCGCC	Overexpression NarT, pMK, and pMHnarT
narOrbsXbaIdir	AAAATCTAGAGGAGGTGCGCCATGTCCA	Overexpression NarO, pMK, and pMHnarO
narOstopEcoRIrev	AATTGAATTCTTAGGCCTTCCTGTCCCTCA	Overexpression NarO, pMK, and pMHnarO

Derivatives of the bifunctional plasmids pMK184 (Km^R^) and pMH184 (Hyg^R^) were constructed for the expression *in trans* of the *narO*, *narK*, and *narT* genes. For this purpose, the corresponding genes and their Shine Dalgarno sequence were amplified by PCR with the primers indicated in Table 3 and cloned into the XbaI and EcoRI sites of these plasmids. In these constructs, transcription of the constitutive promoter PslpA that drives the expression of the *kat* or *hyg* cassettes transcribes also the cloned genes, allowing moderate levels of constitutive expression. A scheme of the complementation plasmids is shown in supplemental Fig. S2.

### Nitrite production

To determine the amount of excreted nitrite, cells were grown at 60 °C in a shaker bath to an OD_550_ of 0.3. After the addition of potassium nitrate (20 mM), the cultures were incubated at 60 °C without stirring for an additional 2 h period. After washing thrice with 50 mM sodium phosphate buffer (pH 7.5), cells were resuspended to an OD_550_ of 0.5 in nitrate-free preheated medium and separated into 2 ml aliquots. These were incubated at 70 °C with increasing concentrations of nitrate (0, 0.01, 0.1, 1, 10, and 20 mM) and the nitrite excreted was determined at different times using previously described methods (Snell & Snell, 1949).

### Nitrate and nitrite sensitivity assays

Sensitivity to nitrite and nitrate was assayed on TB plates and liquid medium in the presence of hygromycin B. For plate assays, a bunch of *Thermus* HB27 colonies harboring, either an empty plasmid (pMH184) or a plasmid expressing a MFS transporter (pMH184NarK, pMH184NarT, and pMH184NarO) were picked on liquid TB medium supplemented with hygromicyn (100 μg/ml) and grown at 65 °C until reaching OD_600_ of 0.3. Serial dilutions (1/10) were carried out on the same medium and 10 μL samples of each dilution where dot plotted on TB plates with hygromycin and either KNO_3_ (200 mM) or NaNO_2_ (150 mM). Colony growth was checked after 48 h of incubation at 60 °C.

For liquid assays, three individual colonies transformed with each plasmid were grown overnight on TB with hygromycin (100 μg/ml). Tubes containing 3 ml of the same medium and increasing concentrations of r KNO_3_ (0–500 mM) or NaNO_2_ (0–140 mM) were inoculated to reach an OD_600nm_ of 0.05. After 24 h of incubation at 65 °C under shaking (170 rpm), the final OD _600nm_ was measured.

## Results and discussion

### NarO belongs to a new family of NarK transporters

**Fig. 1:**
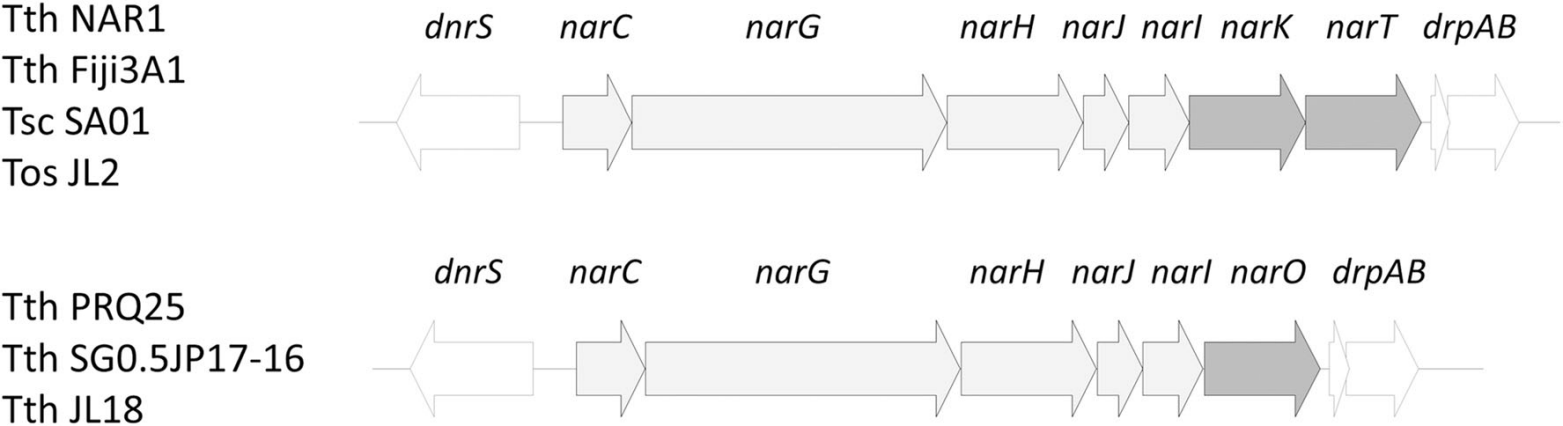
One or two MSF transporters are encoded within the nitrate reductase operon. Organization of the nar operon in the indicated strains of *Thermus* spp.: *T. thermophilus* NAR1, *T. thermophilus* Fiji3A1, *T. oshimai* JL2, *T. scotoductus* SA01, *T. thermophilus* PRQ25, *T. thermophilus* SG0.5JP17-16, and *T. thermophilus* JL18

The *nar* operon from *T. thermophilus* PRQ25, SG0.5JP17-16 and JL18 encodes a single transporter of the NarK family, NarO, instead of the two in tandem transporters of the NarK1 (NarK) and NarK2 (NarT) subfamilies encoded within the *T. thermophilus* strains NAR1, Fiji3A1, *T. oshimae* JL2, and *T. scotoductus* SA01 (Alvarez et al., 2014) (Fig. 1).

**Fig. 2:**
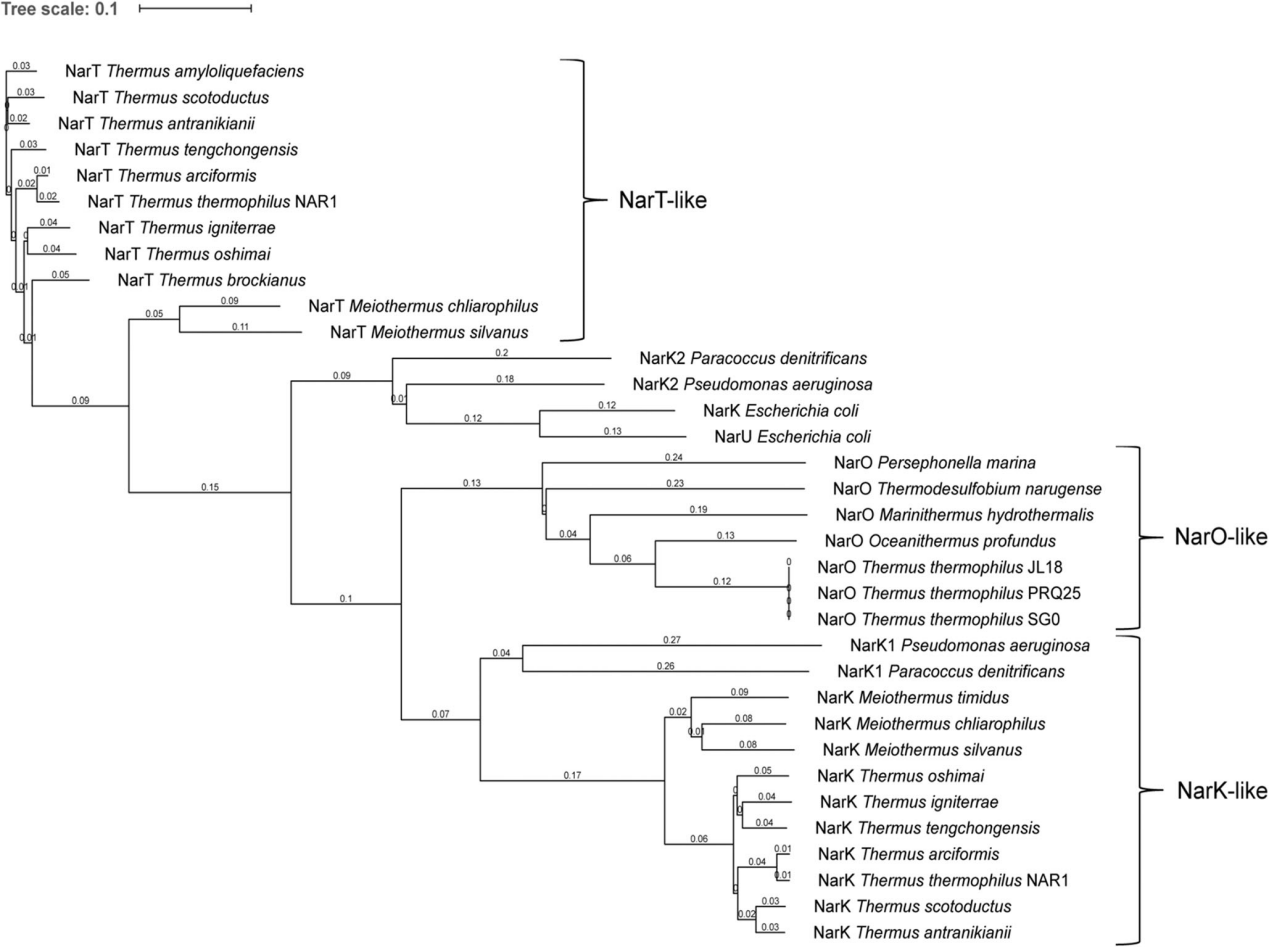
Cladogram of the nitrate/nitrite transporters subfamily. The identification of homologs to the nitrate/nitrite putative transporters was performed by searching with *BLAST* (Altschul et al., 1990) against a non-redundant protein database. The protein data set was aligned using CLUSTAL OMEGA (Sievers et al., 2011). iTOL (Letunic & Bork, 2016) was used for the visualization of the generated tree. Branch-distance is indicated. The proteins used for the comparison are listed in Supplemental Table S1

NarO sequence identities of the PRQ25 strain ranged between 31% with NarK and 27% with NarT of the NAR1 strain, whereas NarK and NarT were 30% identical. Clustal comparisons with *nar*-associated transporters of different bacteria, including *Thermales* and *Proteobacteria* indicate that NarO-like transporters constitute a separate subfamily within the NarK superfamily in addition to those represented by NarK- and NarT-like proteins (Fig. 2).

The presence of in tandem or fused transporters of the MFS family in nitrate respiration clusters of model denitrifying bacteria has been proposed to be related with preferential roles as nitrate/H^+^ symporters for the first protein or protein domain encoded, and as nitrate/nitrite antiporters for the second component (Goddard et al., 2017). In this context, it is interesting to note that the NAR1 and the Fiji3A1 strains are partial denitrifiers that accumulate high concentrations of nitrite extracellularly, whereas the PRQ25 and SG0.5JP17-16 strains are complete denitrifiers that eliminate nitrite from the medium. Therefore, it is tempting to speculate that the presence of two transporters could be more efficient in keeping out nitrite at high concentrations, whereas a single transporter coupled to nitrite respiration could be enough to detoxify for a full denitrifying bacterium.

### The presence of at least one MSF transporter is required for anaerobic growth

To check the hypothesis above in a similar background in the absence of alternative MFS transporters, and also to escape from the low transformation efficiency of *T. thermophilus* PRQ25 (Alvarez et al., 2011; Cava et al., 2008), we constructed two genetically amenable denitrifying derivatives of the aerobic strain HB27 (“Materials and Methods”). One of them contained the *nar* operon from the NAR1 strain (HB27dn), which includes the genes for the NarK and NarT transporters, and the other the *nar* operon from the PRQ25 strain (HB27dp), which contains the gene for the NarO transporter. In addition, both of these strains encode the nitrite respiration cluster (*nic*, from the PRQ25 strain), allowing further detoxification of nitrite. Under aerobic conditions, these two strains grew at similar rates and with similar yield as the aerobic HB27 strain (data not shown), demonstrating that the presence of the denitrification apparatus does not imply a significant burden for aerobic growth.

**Fig. 3:**
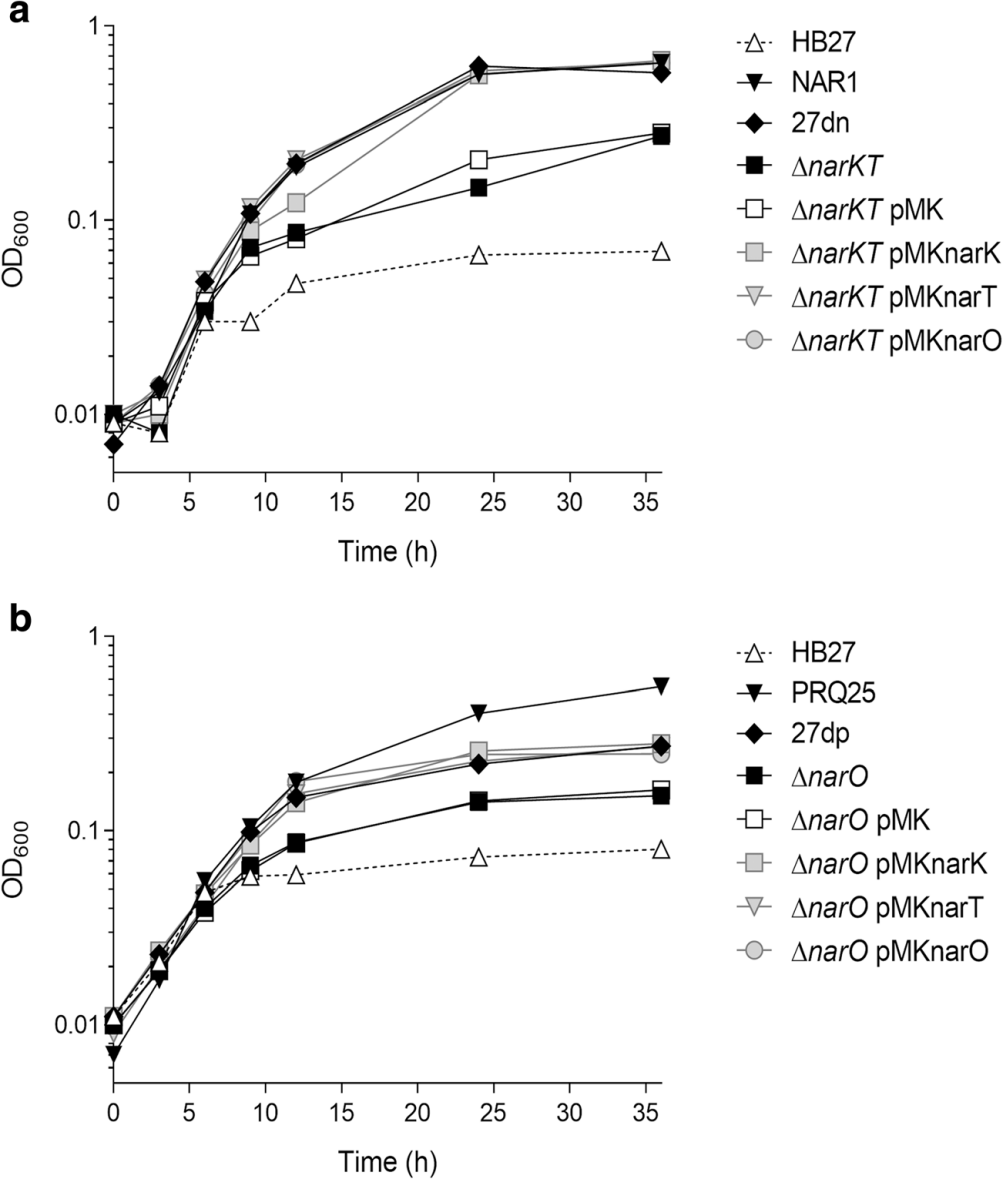
Anaerobic growth with nitrate of the mutants in the nitrate/nitrite transporters. **a** Anaerobic growth curves of strains HB27, NAR1, HB27dn (27dn), HB27dn ∆*narKT* (∆*narKT*), and the HB27dn ∆*narKT* mutant harboring empty plasmid pMK184 (pMK) or overexpressing NarK (pMKnarK), NarT (pMKnarT), or NarO (pMKnarO). **b** Anaerobic growth curves of strains HB27, PRQ25, HB27dp (27dp), HB27dp ∆*narO* (∆*narO*), and the HB27dp ∆*narO* mutant harboring empty plasmid pMK184 (pMK) or overexpressing NarK (pMKnarK), NarT (pMKnarT), or NarO (pMKnarO)

When compared with the native denitrifying strains under nitrate respiration conditions, the HB27dn strain grew also similarly to the NAR1 strain (Fig. 3a), whereas the HB27dp strain grew initially at similar rates as the natural PRQ25 but reaching lower cell yields (Fig. 3b). These data suggest that nitrate respiration shows similar efficiency in the HB27 derivatives as in the parental strain, but that further steps of the denitrification (reduction of nitrite to N_2_O) do not provide the same energy in these lab-generated HB27-derived strains as in the natural PRQ25 strain. As expected, the parental HB27 strain stopped growing after the residual oxygen present in the medium was consumed.

The analysis of the relevance of the transporters was studied by comparison of the growth of these two strains with that of deletion mutants lacking them. For this, a HB27dp Δ*narO* single mutant and a HB27dn Δ*narKT* double mutant were isolated by homologous recombination (Materials and Methods). In both cases, growth under anaerobic conditions with nitrate was greatly impaired with growth yields of 23 and 63% of the Δ*narKT* and Δ*narO* mutants respect to their parental HB27dn and HB27dp strains after 24 h of incubation (Fig. 3a, b ). Interestingly, when either NarO, NarK, or NarT were expressed *in trans* from a plasmid in the single or in the double mutant, the complemented strains were able to grow anaerobically with nitrate in a similar way as their parental strain. As anaerobic growth with nitrate requires both the entrance of nitrate and the extrusion of nitrite, these data suggest that the three proteins can function as nitrate/nitrite transporters under these experimental conditions.

### NarO can transport nitrate and nitrite

**Fig. 4:**
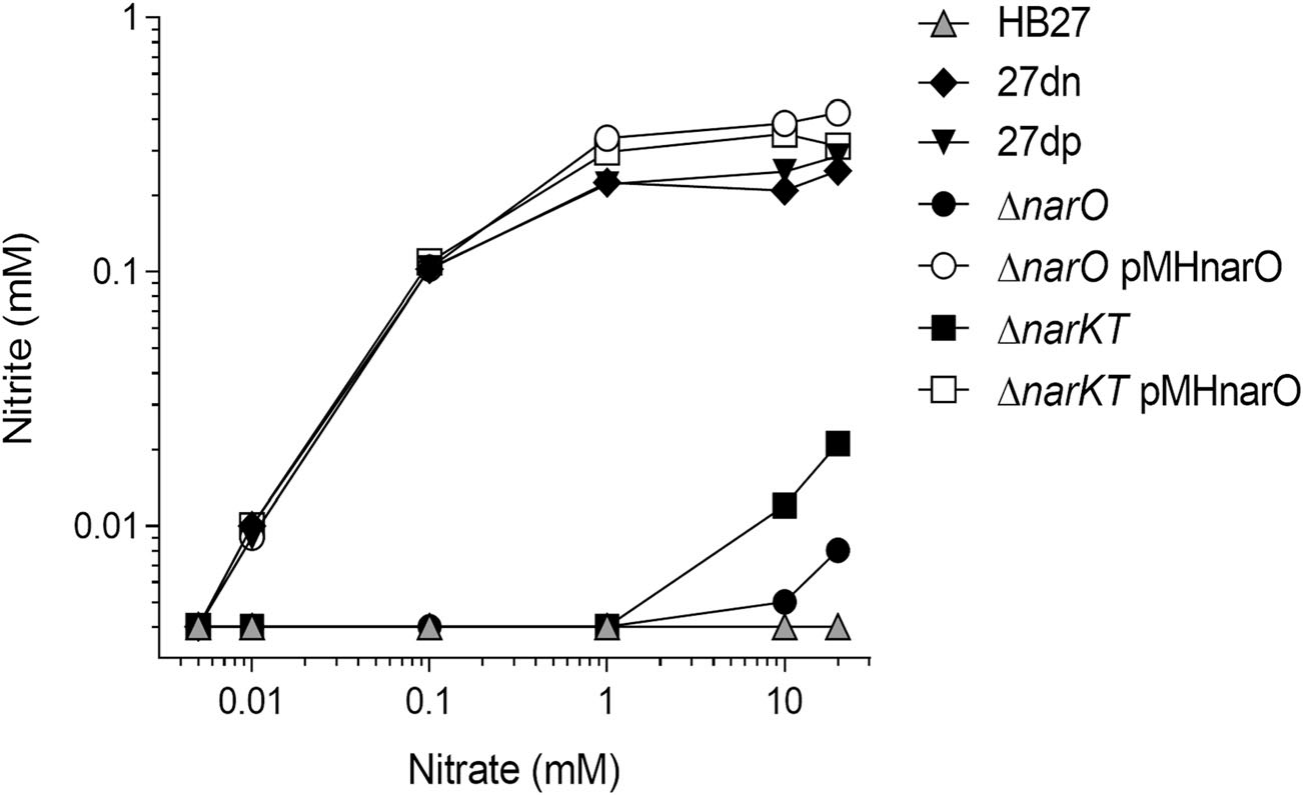
NarO transports both nitrate and nitrite. Nitrite production (mM) in the different strains after 20 min of incubation in the presence of different concentrations of nitrate (0–20 mM). Symbols: ▲ HB27; ♦ 27dn; ▼ 27dp; ● 27dp ∆*narO*; ○ 27dp ∆*narO* pMHnarO; ■ 27dn ∆*narKT*; □ 27dn ∆*narKT* pMHnarO

To determine whether the defective anaerobic growth in the mutants was due to a defect in nitrate and/or nitrite transport, cells treated for 2 h under anoxic conditions with nitrate 20 mM were washed and resuspended in nitrate-free medium. Aliquots were incubated at 70 °C in preheated medium with different concentrations of nitrate, and the nitrite excreted to the supernatant was measured after 20 min (Fig. 4). As it can be observed, HB27dp ∆*narO* and HB27dp ∆*narKT* mutants did not excrete nitrite when low nitrate concentrations (< 1 mM) were present in the medium. Only when the external concentration of nitrate reached 10 mM was a little amount of nitrite (< 0.05 mM) detected in the medium, likely due to leakage. By contrast, their parental HB27dn and HB27dp counterparts efficiently secreted nitrite, supporting the full conversion of nitrate to nitrite and the efficient transport of both nitrate and nitrite. Above 1 mM nitrate, the amount of nitrite secreted remained constant suggesting that either the transporters or the enzyme or both were saturated. Interestingly, the expression of NarO from a plasmid was able to recover the nitrite extrusion rate not only in the HB27dp ∆*narO* background as it could be expected, but also in the HB27dn ∆*narKT* double mutant strain.

These data clearly show that at least under the conditions assayed, the NarO protein is as efficient as nitrate/nitrite transporter (antiporter) as the combination of NarK and NarT.

### Protection provided by the MFS transporters against nitrate and nitrite toxicity

**Fig. 5:**
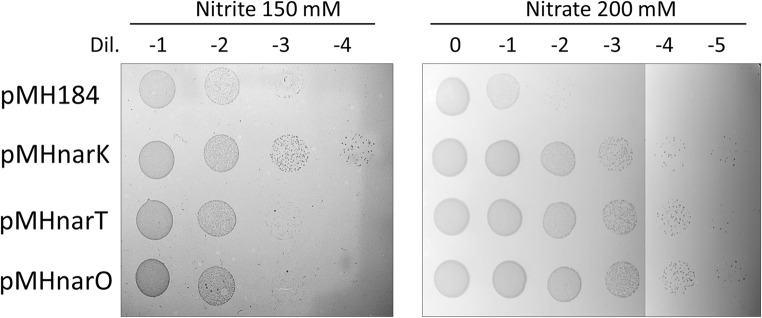
Resistance to nitrite and nitrate provided by MFS transporters on plate assays. Dot plate assays with serial 1:10 dilutions of cultures of *T. thermophilus* HB27 transformed with the indicated plasmids. TB plates with hygromycin B containing sodium nitrite or potassium nitrate 150 and 200 mM, respectively, were used

All the above results support for the three subtypes of transporters a role as nitrate and nitrite antiporters in denitrifying strains under the conditions assayed (Figs. 3 and 4). In order to check the putative role in warding off against toxicity of the resulting nitrogen oxides in a context in which biological conversion by reduction is minimal, we expressed the three genes in the aerobic strain *T. thermophilus* HB27 and compared the resistance to nitrate (200 mM) and nitrite (150 mM) of the transformants with that provided by an empty plasmid. As can be observed in the qualitative resistance assay on plates shown in Fig. 5, expression of NarK produced resistance to nitrite, allowing the transformant to grow much better (around 100-fold) than the strain with the empty plasmid. By contrast, expression of NarT or NarO did not result in a significant increased resistance to the assayed concentration of nitrite in these plate-based growth assays. When the same strains were plated onto plates containing 200 mM of nitrate, all the three proteins produced a 1000-fold increase in resistance compared with the strain carrying the empty plasmid.

**Fig. 6:**
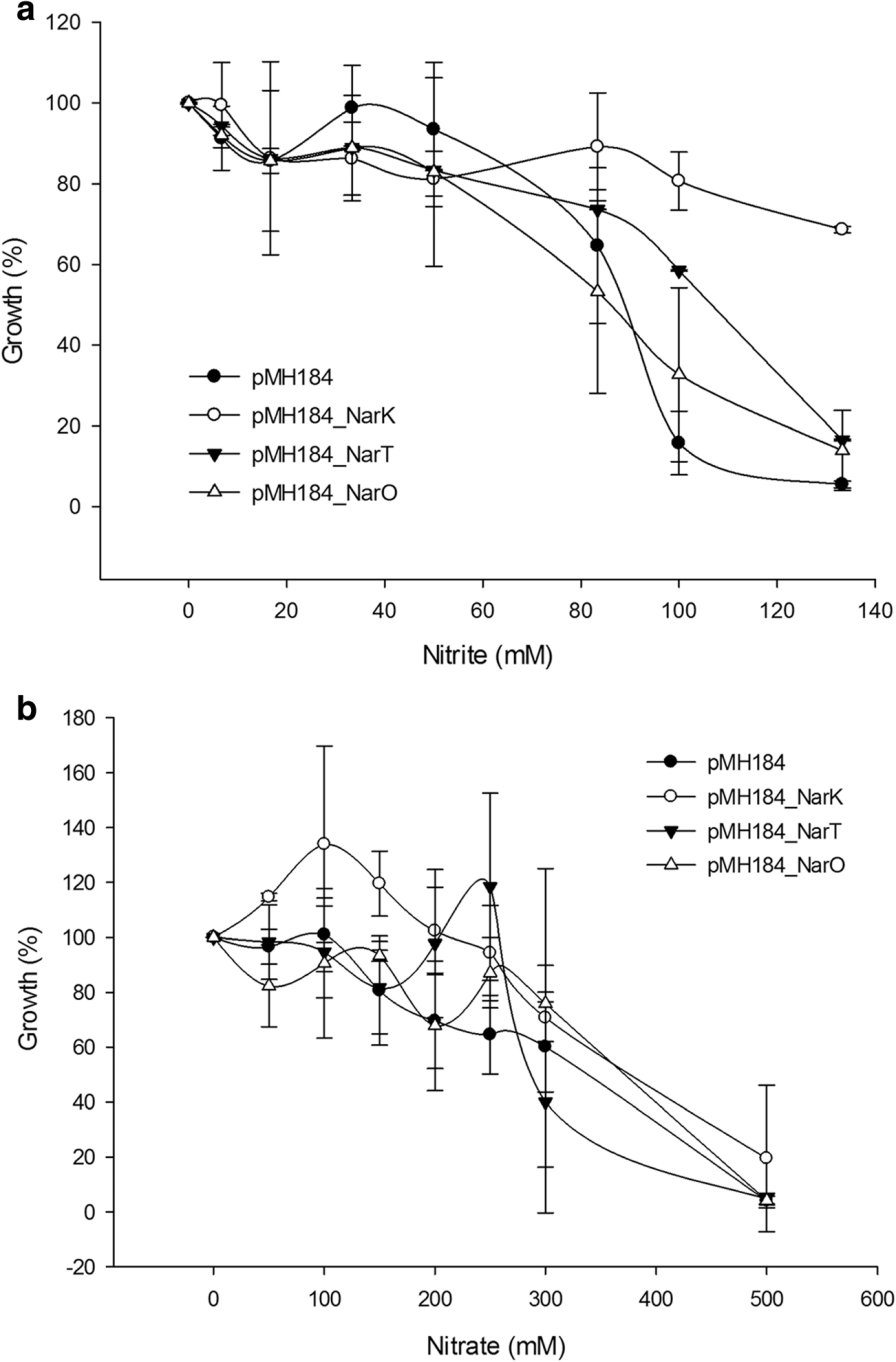
Liquid growth inhibition by nitrite and nitrate. Cultures of the aerobic *T. thermophilus* HB27 strain transformed with the indicated plasmid were grown for 24 h at 65 °C on TB medium with hygromycin (100 μg/ml) in the presence of the indicated concentrations of nitrite (**a**) or nitrate (**b**). The mean percentage of growth (OD_600_) of three different colonies respect to the controls without nitrogen oxide is represented

To evaluate these resistances in liquid medium in a more quantitative manner, identical amounts of cells of the HB27 strain transformed with the plasmids expressing each of the transporters and with the empty plasmid were inoculated in TB medium with increasing concentrations of nitrite, allowing them to grow for 24 h at 65 °C. The results of Fig. 6a show that expression of NarK from a plasmid allows the cell to grow above 120 mM of nitrite, a concentration at which the wild type with the empty plasmid cannot grow at all. It is also interesting to note that NarT and NarO provide some resistance to lower nitrite concentrations. Thus, we concluded that in addition to the role as nitrate/nitrite antiporters, these three proteins, and specially NarK, can work as efficient nitrate extrusion pumps under the conditions assayed.

Results with nitrate in similar experiments supported that the three proteins also provide resistance to nitrate at a critical concentration between 200 and 300 mM, suggesting that the proteins could also pump out nitrate (Fig. 6b).

## Conclusions

Previous work in *T. thermophilus* showed that absence of NarT (NarK2 subfamily) had more severe effects at the levels of anaerobic growth rate, nitrate transport, and extracellular nitrite accumulation than the absence of NarK in the nitrate-respiring NAR1 strain (Ramirez et al., 2000). Through a series of analyses, we concluded that despite both proteins being able to function as nitrate/nitrite antiporters, NarK showed preference for nitrate transport and NarT for nitrite extrusion. A similar conclusion was further reached by other groups working on the NarK1 and NarK2 transporters of *Pseudomonas aeruginosa* PA01 (Sharma et al., 2006) and the N- and C-terminal domains of the double-NarK fusion protein of *Paracoccus denitrificans* (Goddard et al., 2008). In this work, we wondered why in several denitrifying strains of *T. thermophilus* a single transporter of a different MFS family was present. For this, we isolated mutants lacking any nitrate/nitrite transporters in denitrifying derivatives of the HB27 strain that were genetically amenable proxies of the natural nitrate-respiring (NAR1) and nitrate-denitrifying (PRQ25) strains. We showed that these mutants lacking nitrate/nitrite transporters were defective in anaerobic growth with nitrate and that expression of any of the three classes of transporters, NarK, NarT, or NarO, was capable of complementing the anaerobic growth. Therefore, NarO, the new class of transporter, functions efficiently as nitrate/nitrite antiporter being able to replace the NarK-NarT two transporter system found in many *nar* operon of *T. thermophilus*.

A putative advantage of having two transporters over a single one could be related to an increased efficiency in nitrite detoxification in partial denitrifying isolates. In this sense, it is interesting to note that the expression of these transporters in a strict aerobic genetic context, in which reduction of nitrite or nitrate was not possible, allowed us to identify NarK as a good provider of resistance to high levels of nitrite. In contrast to previous hypothesis that suggested a major role as nitrate transporter, these data support that NarK is a very efficient nitrite extrusion protein, a role that cannot be performed efficiently by NarT or NarO which could be better as nitrate/nitrite antiporters.

A yet difficult to understand question is the resistance to nitrate provided by the overexpression of the three transporters to the aerobic strain. As nitrite cannot be produced in significant amounts in this aerobic context, and high resistance to nitrite is only provided by NarK, the most likely explanation for these results is that the three enzymes can also function as nitrate extrusion proteins in the absence of nitrite. Therefore, our data suggest that these MFS subfamilies are actually proton: nitrate/nitrite antiporters that have further been adapted to transport nitrate in denitrifying strains.

## Electronic supplementary material

Fig. S1 (PNG 71 kb)

High resolution image (TIF 3475 kb)

Fig. S2 (PNG 120 kb)

High resolution image (TIF 2711 kb)

ESM 1 (DOCX 19 kb)
