# Supplementary figures and images for: A new family of nitrate/nitrite transporters involved in denitrification
Source: Int Microbiol. 2018 Jul 20;22(1):19–28. doi: 10.1007/s10123-018-0023-0 (PMC6394727; doi:10.1007/s10123-018-0023-0)

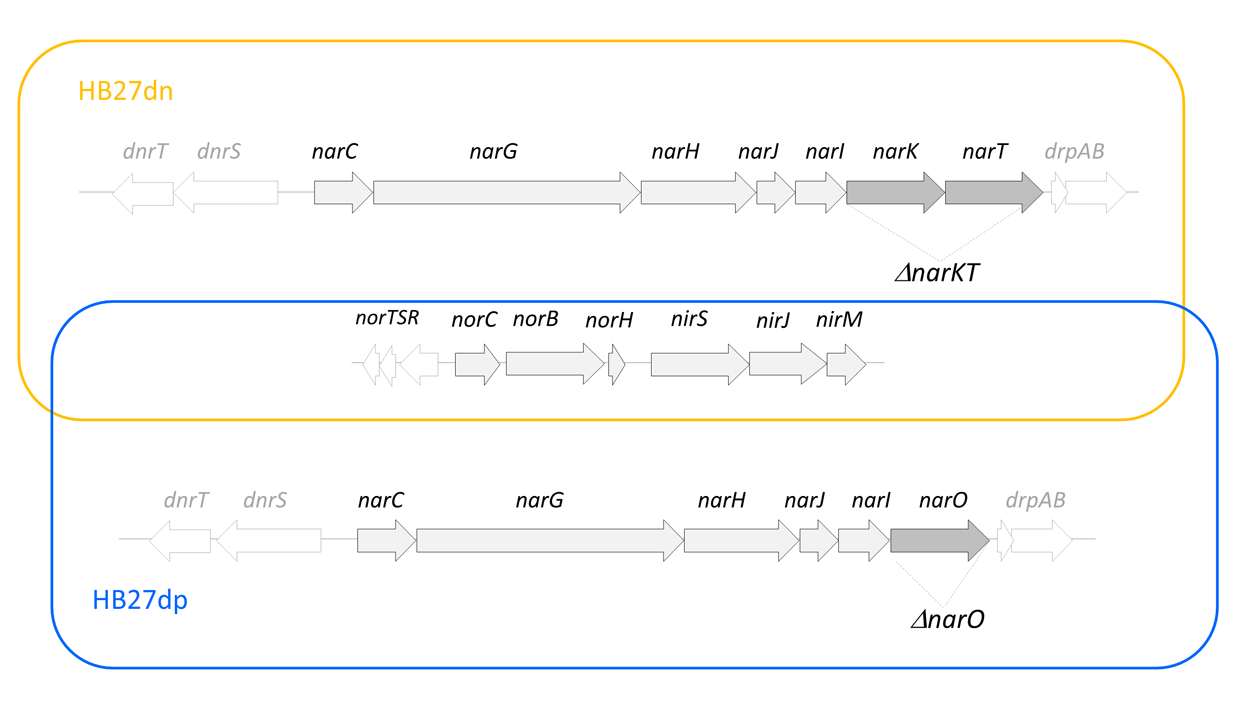

Supplement: Supplementary file 1 — (PNG 71 kb) [file 10123_2018_23_Fig7_ESM.png]

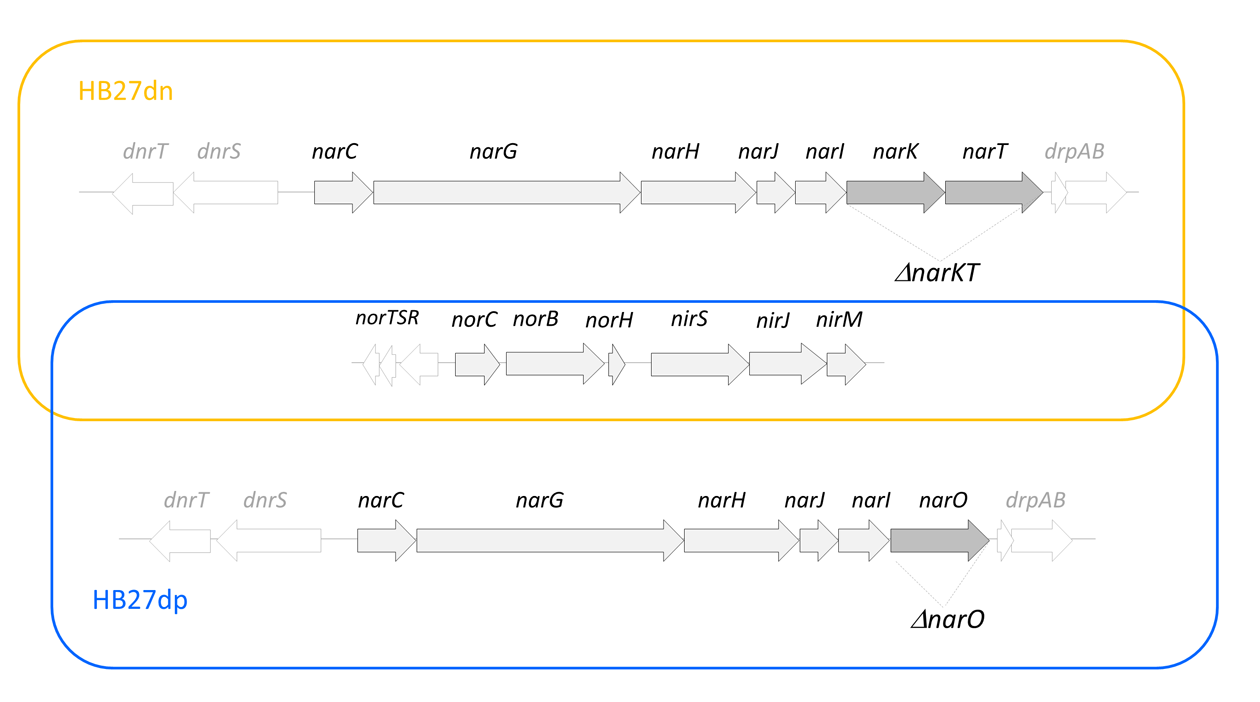

Supplement: Supplementary file 2 — High resolution image (TIF 3475 kb) [file 10123_2018_23_MOESM1_ESM.tif]

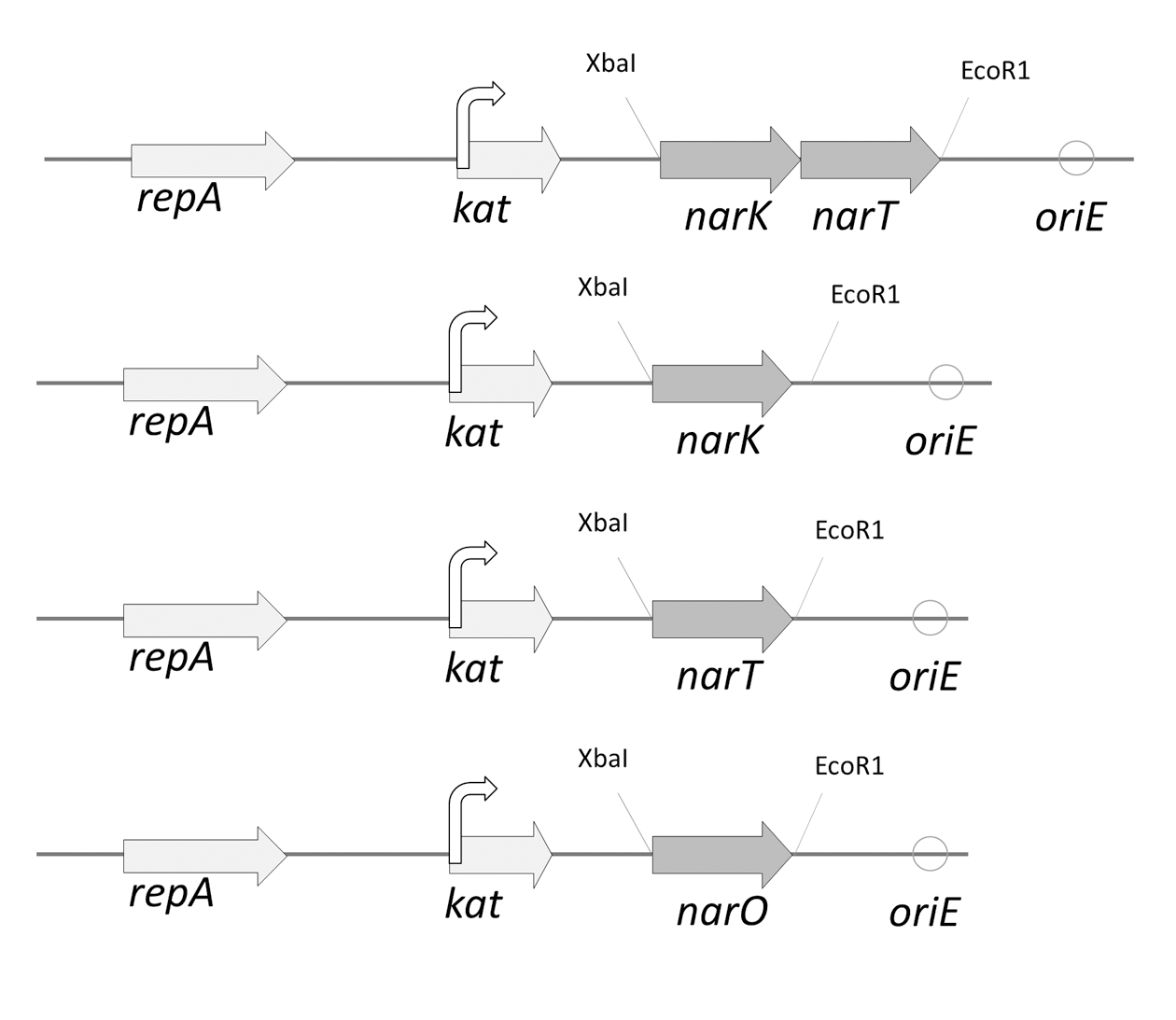

Supplement: Supplementary file 3 — (PNG 120 kb) [file 10123_2018_23_Fig8_ESM.png]
