# Supplementary material for: A new family of nitrate/nitrite transporters involved in denitrification
Source: Int Microbiol. 2018 Jul 20;22(1):19–28. doi: 10.1007/s10123-018-0023-0 (PMC6394727; doi:10.1007/s10123-018-0023-0)
Supplement: Supplementary file 5 — (DOCX 19 kb) [file 10123_2018_23_MOESM3_ESM.docx]

**Supplemental material**

**Figure S1. Denitrification clusters in the HB27dn and HB27dp denitrifying derivatives of the aerobic strain HB27 and its transporter deficient mutants.** The clusters involved in denitrification are depicted. HB27dn contains the *nar* operon from the NAR1 strain, containing two transporters of the Nark family (dark grey). The HB27dp derivative contains the *nar* operon from the PRQ25 strain with a single transporter of the NarO subtype (dark grey). Both strains share the nitrite respiration cluster of the PRQ25 strain. Deletion of the transporters allows for the selection of the indicated mutants.

**Figure S2. Scheme of the plasmids used in complementation experiments.** Cartoon shows the scheme of the pMK184 or pMH184 derivative plasmids used for complementation experiments The pMH184 derivative plasmids carry the *kat* cassette conferring resistance to Kanamycin, while the pMH184 derivative plasmids carry the *hph*5 gene conferring resistance to Hygromycin.

**Table S1. Proteins used for the phylogenetic comparisons in Figure 2.**

| Reference sequence | NP_415741.1 | NP_415986.1 | AEB12575.1 | WP_027891934.1 | WP_084542339.1 | ADH62190.1 | ADH62189.1 | WP_018467781.1 | ADR36881.1 | ABL72301.1 | ABL72301.1 | ACO03221.1 | NP_252566.1 | WP_079453567.1 | AEE13746.1 | WP_038057440.1 | WP_028493910.1 | WP_028493911.1 | SDE92861.1 | SDE92886.1 | APD08620.1 | WP_026175164.1 | WP_018112082.1 | AFV76087.1 | AFV76086.1 | ADW22361.1 | ADW22360.1 | WP_038041973.1 | WP_038041974.1 | AFH40157.1 | CAB65479.2 | CAB65480.2 |  | AEG34573.1 |
| --- | --- | --- | --- | --- | --- | --- | --- | --- | --- | --- | --- | --- | --- | --- | --- | --- | --- | --- | --- | --- | --- | --- | --- | --- | --- | --- | --- | --- | --- | --- | --- | --- | --- | --- |
| Description | nitrate/nitrite transporter [*Escherichia coli* str. K-12 substr. MG1655] | nitrate/nitrite transporter [*Escherichia coli* str. K-12 substr. MG1655] | major facilitator superfamily MFS_1 [*MariniThermus hydrothermalis* DSM 14884] | NarK/NasA family nitrate transporter [Meio*Thermus* chliarophilus] | NarK/NasA family nitrate transporter [Meio*Thermus chliarophilus* | major facilitator superfamily MFS_1 [Meio*Thermus silvanus* DSM 9946] | major facilitator superfamily MFS_1 [Meio*Thermus silvanus* DSM 9946 | NarK/NasA family nitrate transporter [Meio*Thermus timidus*] | major facilitator superfamily MFS_1 [Oceani*Thermus profundus* DSM 14977] | nitrite transporter [*Paracoccus denitrificans* PD1222] | nitrite transporter [*Paracoccus denitrificans* PD1222] | major facilitator family transporter [*Persephonella marina* EX-H1] | nitrite extrusion protein 1 [*Pseudomonas aeruginosa* PAO1] | MFS transporter [*Pseudomonas aeruginosa*] | major facilitator superfamily MFS_1 [*Thermodesulfobium narugense* DSM 14796] | NarK family nitrate/nitrite MFS transporter [*Thermus amyloliquefaciens*] | NarK/NasA family nitrate transporter [*Thermus antranikianii*] | NarK family nitrate/nitrite MFS transporter [*Thermus antranikianii*] | MFS transporter, NNP family, nitrate/nitrite transporter [*Thermus arciformis*] | MFS transporter, NNP family, nitrate/nitrite transporter [*Thermus arciformis*] | Nitrate/nitrite transporter NarK [*Thermus brockianus*] | NarK/NasA family nitrate transporter [*Thermus igniterrae]* | NarK family nitrate/nitrite MFS transporter [*Thermus igniterrae*] | nitrate/nitrite transporter [*Thermus oshimai* JL-2] | nitrite extrusion protein, nitrite facilitator [*Thermus oshimai* JL-2] | NarK1 protein [*Thermus scotoductus* SA-01] | nitrite extrusion protein 1 [*Thermus scotoductus* SA-01] | NarK/NasA family nitrate transporter [*Thermus tengchongensis*] | NarK family nitrate/nitrite MFS transporter [*Thermus tengchongensis*] | nitrate/nitrite transporter (plasmid) [*Thermus thermophilus* JL-18] | Nitrate/nitrite antiporter [*Thermus thermophilus* HB8] | Nitrate/nitrite antiporter [*Thermus thermophilus* HB8] |  | major facilitator superfamily MFS_1 (plasmid) [*Thermus thermophilus* SG0.5JP17-16] |
| Protein | NarK_Escherichia_coli | NarU_Escherichia_coli | NarO_Marinithermus_hydrothermalis | NarK_Meiothermus_chliarophilus | NarT_Meiothermus_chliarophilus | NarK_Meiothermus_silvanus | NarT_Meiothermus_silvanus | NarK_Meiothermus_timidus | NarO_Oceanithermus_profundus | NarK1_Paracoccus_denitrificans | NarK2_Paracoccus_denitrificans | NarO_Persephonella_marina | NarK1_Pseudomonas_aeruginosa | NarK2_Pseudomonas_aeruginosa | NarO_Thermodesulfobium_narugense | NarT_Thermus amyloliquefaciens | NarK_Thermus_antranikianii | NarT_Thermus_antranikianii | NarK_Thermus_arciformis | NarT_Thermus_arciformis | NarT_Thermus_brockianus | NarK_Thermus_igniterrae | NarT_Thermus_igniterrae | NarK_Thermus_oshimai | NarT_Thermus_oshimai | NarK_Thermus_scotoductus | NarT_Thermus_scotoductus | NarK_Thermus_tengchongensis | NarT_Thermus_tengchongensis | NarO_Thermus_thermophilusJL18 | NarK_Thermus_thermophilusNAR1 | NarT_Thermus_thermophilusNAR1 | NarO_Thermus_thermophilusPRQ25 | NarO_Thermus_thermophilusSG0 |

**Pro**
